# Exploring Attitudes Toward MDMA-Assisted Therapy in Mental Health Care

**DOI:** 10.1192/j.eurpsy.2026.11674

**Published:** 2026-07-31

**Authors:** A. Mechlińska, D. Swieczkowski, A. Kwaśny, M. Pastuszak, W. J. Cubała

**Affiliations:** 1 Department of Psychiatry; 2 Department of Toxicology, Medical University of Gdańsk, Gdańsk; 3 Department of Clinical Research and Development, LUXMED Group, Warsaw, Poland

## Abstract

**Introduction:**

MDMA (3,4-methylenedioxymethamphetamine)–assisted therapy has gained growing scientific attention as a potential treatment for post-traumatic stress disorder and other mental health conditions. Although clinical studies suggest potential benefits, concerns remain regarding cardiovascular safety, methodological rigor, and variability in psychotherapeutic support (Singh *et al*. CNS Drugs 2025; 4 339-343). Ethical and legal uncertainties further limit its acceptance in clinical practice. Exploring healthcare professionals’ attitudes toward this emerging therapy may help identify barriers to its safe and evidence-based implementation.

**Objectives:**

The purpose of this survey is to gather the opinions of healthcare professionals regarding the potential use of MDMA-assisted therapy (3,4-methylenedioxymethamphetamine) in clinical practice, as well as to identify possible barriers to its implementation.

**Methods:**

A cross-sectional survey was conducted among 40 healthcare professionals (psychiatrists, psychiatry residents, and psychotherapists representing various therapeutic approaches) to assess their attitudes towards MDMA-assisted therapy. The anonymous online questionnaire included demographic questions, self-assessment of knowledge about MDMA, and 12 items evaluating attitudes toward its therapeutic use and potential risks.

**Results:**

An equal number of psychotherapists (in total) and psychiatry residents participated in the study (47.5%), while only 10% of the respondents were board-certified psychiatrists (See Figure 1). The majority worked in general hospitals with psychiatric wards or private practice. Although 95% had no direct research involvement with MDMA, 87.5% supported further scientific studies on its therapeutic potential. Despite the fact that most respondents reported limited knowledge regarding the therapeutic use of MDMA, 67.5% believed that treatment with this substance carries risks even when conducted under medical supervision. Over 60% recognized possible positive effects of MDMA in treating mental disorders, especially when combined with psychological support, which was perceived as increasing both safety and efficacy (See Figure 2). The main barriers identified were legal regulations (60%), lack of treatment standards (60%), and insufficient training or knowledge (60%) (See Figure 3).

**Conclusions:**

This study highlights a cautious optimism among healthcare professionals toward MDMA-assisted psychotherapy. Enhancing education, promoting further research, and establishing clear legal and clinical frameworks may facilitate its safe application in mental health care.

**Disclosure of Interest:**

A. Mechlińska: None Declared, D. Swieczkowski : None Declared, A. Kwaśny Grant / Research support from: Grants: Beckley Psytech, Compass Pathways, GH Research, MSD., M. Pastuszak Grant / Research support from: Grant / Research support from: Beckley Psytech, Compass Pathways, GH Research, MSD, W. Cubała Grant / Research support from: Wiesław Jerzy Cubała: i) Grants: Acadia, Alkermes, Allergan, Angelini, Auspex Pharmaceuticals, Beckley Psytech, BMS, Celon, Cephalon, Compass Pathways, Cortexyme, Ferrier, Forest Laboratories, GedeonRichter, GH Research, GWPharmaceuticals, HMNC Brain Health, IntraCellular Therapies, Janssen, KCR, Lilly, Lundbeck, Minerva, MSD, NIH, Neumora, Novartis, Orion, Otsuka, Recognify Life Sciences, Sanofi, Servier Honoraria: Adamed, Angelini, AstraZeneca, BMS, Celon, GH Research, GSK, Janssen, KRKA, Lekam, Lundbeck, Minerva, NeuroCog, Novartis, Orion, Pfizer, Polfa Tarchomin, Sanofi, Servier, Zentiva Advisory boards: Angelini, Celon (ended 2021), Douglas Pharmaceuticals, GH Research, Janssen, MSD, Novartis, Polpharma, Sanofi

